# Effect and mechanism of longkui yinxiao soup in treating psoriasis in mice

**DOI:** 10.3389/fphar.2023.1136604

**Published:** 2023-03-13

**Authors:** Congcong Zhu, Ya Chen, Zongguang Tai, Huijun Pan, Min Shen, Zhongjian Chen, Quangang Zhu

**Affiliations:** ^1^ Shanghai Skin Disease Hospital, School of Medicine, Tongji University, Shanghai, China; ^2^ Shanghai Engineering Research Center for Topical Chinese Medicine, Shanghai, China

**Keywords:** inflammation, keratinocyte proliferation, longkui yinxiao soup, RAP1-MAPK signaling inflammation, RAP1-MAPK signaling, psoriasis

## Abstract

**Objective:** Longkui Yinxiao Soup is a traditional Chinese medicine formula used to treat psoriasis for decades. Although Longkui Yinxiao Soup showed promising efficacy in clinical practice, the regulatory mechanisms of Longkui Yinxiao Soup remain elusive. This study aimed to explore the underlying mechanisms of Longkui Yinxiao Soup in a psoriasis-like mouse model.

**Methods:** Longkui Yinxiao Soup was quality controlled by determining the contents of imperatorin and rhoifolin using high-performance liquid chromatography. The imiquimod-induced psoriasis-like mouse model was used to study the therapeutic effect and mechanism of Longkui Yinxiao Soup. The histopathological skin changes were observed by hematoxylin and eosin staining; the infiltration of proliferating proteins, proliferating cell nuclear antigen and Ki67, in skin tissues were observed by immunohistochemical analysis; and the inflammatory factors such as interleukin (IL)-6, tumor necrosis factor (TNF)-α, IL-23, and IL-17 in serum were detected using enzyme-linked immunosorbent assay. RNA sequencing and bioinformatic analysis were used to predict the mechanism of LYS against psoriasis. mRNA expressions of p38, extracellular regulated protein kinases (ERK), mitogen-activated protein kinase 3 (MEK3), mitogen-activated protein kinase 6 (MEK6), RAP1 GTPase activating protein (Rap1gap), and Rap1 were determined using real-time quantitative polymerase chain reaction. The expression levels of proteins related to Rap1–mitogen-activated protein kinase signaling pathways were measured by Western blotting.

**Results:** A quality-control method for Longkui Yinxiao Soup was successfully established using imperatorin and rhoifolin as content determination indexes. Longkui Yinxiao Soup significantly ameliorated the psoriatic symptoms in mice. The serum levels of inflammatory cytokines such as IL-6, TNF-α, IL-23, and IL-17 were decreased, and the expression levels of antigen identified by monoclonal antibody Ki67 (Ki67) and PCNA in skin tissues were downregulated. Moreover, the inhibition of Rap1–MAPK signaling pathways by Longkui Yinxiao Soup was detected.

**Conclusion:** This study confirmed the antipsoriatic activity of Longkui Yinxiao Soup in psoriasis-like mice. This might be due to the inhibition of inflammatory factor secretion, keratinocyte proliferation, and the Rap1–MAPK signal pathway.

## 1 Introduction

Psoriasis is a common, chronic papulosquamous skin disease characterized by the development of erythematous, indurated, scaly, pruritic, and often painful skin plaques (Korman, 2020; Griffiths et al., 2021). The etiology of psoriasis is not yet clear. Studies reported that genetic and environmental factors were the key components. Epidemiological studies provided evidence that psoriasis affected about 3% of the United States population and approximately 125 million people worldwide. Further, psoriasis increases the risk of depression, anxiety, and inflammatory bowel disease and imposes an immense burden on patients and society (Schonmann et al., 2019; Walter, 2022).

Excessive proliferation and impaired differentiation of keratinocytes are typical histopathological features of psoriasis. The progression of psoriasis is triggered by the upregulation of pro-inflammatory cytokines resulting from the activation of keratinocytes and immune cells (Shi et al., 2019; Kamata and Tada, 2022; Ortiz-Lopez et al., 2022). Dendritic cells secrete IL-23, IL-6, and TNF-α after abnormal activation, while Th17 cells induced by IL-23 produce inflammatory cytokines such as IL-17A, IL-22, TNF-α, and IL-6 (Sharma et al., 2022). The IL-23/Th17 axis is considered closely related to the occurrence and maintenance of psoriasis. These inflammatory factors work together to promote keratinocyte proliferation and other symptoms of psoriasis.

Although modern medicine makes remarkable progress in treating psoriasis by developing drugs such as glucocorticoids, tretinoin, methotrexate, and biological preparations, the long-term use of these drugs may cause various adverse effects (Armstrong and Read, 2020). Therefore, traditional Chinese medicine as an alternative and effective therapy deserves further study.

Longkui Yinxiao Soup (LYS) has been used as an in-hospital formulation for psoriasis vulgaris in the Shanghai Skin Disease Hospital for more than 30 years. LYS contains five botanical drugs (Table 1), including *Solanum nigrum* L. (Chinese name: Longkui), *Pteris multifida* Poir. (Chinese name: Fengweicao), *Arnebia euchroma* (Royle) Johnst. (Chinese name: Zicao), *Angelica dahurica* (Fisch.ex Hoffm.) Benth.et Hook.F. (Chinese name: Baizhi), and *Dioscorea opposita* Thunb. (Chinese name: Shanyao). In clinical observations of LYS treatment, the total effective rate of LYS and Avia capsule combination therapy was 87.50%, and the total effective rate of therapy using Avia capsule in psoriasis vulgaris was 70.00% (Zhao et al., 2022). LYS has the ability to clear heat, cool blood, remove toxins, and nourish the body, indicating its effectiveness in reducing the level of inflammation in the body. As an immune disease caused by heat and toxins in the blood, psoriasis can be treated using a decoction that releases heat and toxins by reducing inflammation. Although the clinical efficacy of LYS is clear and widely recognized by physicians and patients, the lack of quality standards and advanced studies on the mechanism of action hinder its verification and confirmation as an effective, scientifically proven drug for the treatment of psoriasis.

**TABLE 1:** Information of components in LYS.

Latin name	English name	Chinese	Plant part	Amount g)
*Solanum nigrum* L	Black Nightshade	Longkui	Herb	2,700
*Pteris multifida* Poir	Herba Pteridis Multifidae	Fengweicao	Leaf	1,350
*Arnebia euchroma* (Royle) Johnst	Arnebiae Radix	Zicao	Root	1,350
*Angelica dahurica* (Fisch.ex Hoffm.) Benth.et Hook.F	Angelicae Dahuricae Radix	Baizhi	Root	810
*Dioscorea opposita* Thunb	Dioscoreae Rhizoma	Shanyao	Rhizome	810

In this study, we performed the quality control of LYS and then evaluated its antipsoriatic effect in the imiquimod (IMQ)-induced mouse model of psoriasis by regulating keratinocyte proliferation and inflammatory factor secretion. Finally, RNA sequencing and molecular biology methods were adopted to predict the potential target of LYS. This study aimed to clarify the mechanism of the effective substances in LYS inducing efficacy and blocking the pathogenesis of psoriasis.

## 2 Materials and methods

### 2.1 Chemical and reagents

LYS was obtained from Shanghai Baolong Pharmaceutical Co., Ltd. (Shanghai, China). IMQ cream was purchased from Sichuan Mingxin Pharmaceutical Co., Ltd. (Sichuan, China). Imperatorin (purity ≥98%) and rhoifolin (purity ≥98%) were purchased from the National Institutes for Food and Drug Control of China (Beijing, China). All primers were synthesized by Sangon Biotech (Shanghai, China). Antibodies specific to MEK3 + MEK6 (ab200831) and p38 delta/mitogen-activated protein kinase (MAPK) 13 + p38 alpha/MAPK14 (ab31828) were obtained from Abcam Inc. (Cambridge, United Kingdom), and Rap1gap (DF4383) was obtained from Affinity Biosciences Inc. (OH, United States). Ki67 rabbit monoclonal antibody (AF1738), proliferating cell nuclear antigen (PCNA) rabbit monoclonal antibody (AF1363), phospho-p38 MAPK (Thr180/Tyr182) (AM063) antibody, Horseradish Peroxidase (HRP)-labeled goat anti-rabbit immunoglobulin G (IgG) (H + L) (A0208), and HRP-labeled goat anti-mouse IgG (H + L) (A0216) were purchased from Beyotime Biotechnology (Shanghai, China). The enhanced chemiluminescence reagent was obtained from Yeasen (Shanghai, China).

### 2.2 Animal participants

Male BALB/c mice (22 ± 2 g) aged 6–8 weeks, purchased from Shanghai JieSiJie Laboratory Animals Co., Ltd. (Shanghai, China), were used in the experiments. The mice were offered free access to food and water within a specific pathogen-free environment. The use of animal participants in this study was reviewed and approved by the Care and Use Committee of the Shanghai Skin Disease Hospital (ethics clearance number: 2022–60.)

### 2.3 Preparation of LYS

The five botanical drugs included in LYS (Table 1) were purchased from Dechang Pharmaceutical Co., Ltd. (Anhui, China). For preparing the LYS, the crude drugs were crushed into small pieces. They were added into 10 times (*v*/*w*) purified water for 2 h and then decocted in 8 times water (*v*/*w*) for 1 h. The decoction was then filtrated, and the filtrate was collected. The filtrate obtained after two times of filtration was mixed and concentrated at 80°C to a relative density of 1.27.

### 2.4 Quality control of LYS

The analytical method was validated using a Waters Alliance high-performance liquid chromatography (HPLC, MA, United States) system containing an e2695 separation module and a 2,998 photodiode array detector. Both imperatorin (active ingredient of Baizhi) and rhoifolin (active ingredient of Fengweicao) were analyzed using an Agilent Eclipse Plus C18 column (4.6 mm × 250 mm, 5 μm). The other chromatographic conditions for HPLC were as follows: the eluent was acetonitrile–water (65:35) for imperatorin and acetonitrile–water (20:80) for rhoifolin; the monitoring wavelength was set to 300 nm for imperatorin and 345 nm for rhoifolin; the column oven temperature was 30°C; the flow rate of the eluent was 1.0 mL min^–1^; and the sample volume was 10 μL.

### 2.5 Animal experiments

The mice were subjected to adaptive feeding for 7 days prior to the initiation of the experiments. Thirty-five mice were randomly categorized into five groups: the control group, the model group (IMQ group), the methotrexate (MTX, 1 mg/kg)-treated group, and two groups treated with LYS intragastric administration at the doses of 4.9 g/kg and 43.8 g/kg of crude drug (LYS-Low and LYS-High) from day 1 to day 12. The low dose of LYS was converted according to human equivalent dosages based on body surface area. The backs of all the mice were shaved and depilated using depilatory cream to expose the back skin. Four groups except the control group received the application of 62.5 mg IMQ cream (5% *w*/*w*) daily on their back skins for five consecutive days. On day 13, the mice were sacrificed after being narcotized by inhalation of diethyl ether, and the blood samples and back skin tissues were collected.

### 2.6 Psoriasis area and severity index as well as spleen index assessments

The body weight of the mice was recorded every day during the experiment. The psoriasis area and severity index (PASI) was used to evaluate the severity of the skin inflammation, and the spleen index was measured on day 13. The degrees of erythema, scaling, and thickening were scored independently on a scale of 0–4: 0 for none; one for slight; two for moderate; three for severe; four for very severe. The severity of the inflammation was measured using the cumulative score (erythema plus scaling plus thickening) on a scale of 0–12. The spleen index was calculated as follows:

Spleen index (mg/g) = Spleen weight (mg)/Mouse weight (g) × 10.

### 2.7 Histopathological analysis

On day 13, the back skin tissues were collected and fixed in 10% buffered formalin and embedded in paraffin. After staining with hematoxylin and eosin (H and E), the samples were examined using an optical microscope (Nikon Eclipse E100, Japan). The epidermal thickness was measured at five randomly selected spots.

### 2.8 Enzyme-linked immunosorbent assay

The serum samples were separated by centrifugation from the blood samples at 12,000*g* and 4°C for 20 min. Then, the levels of the cytokines IL-6 (No. MAN0017508; Invitrogen, CA, USA), TNF-α (No. MAN0017423; Invitrogen), IL-23 (No. 2111–1; Dakewe Biotech, Shenzhen, China) and IL-17 A (No. 2112–2; Dakewe Biotech) were determined using enzyme-linked immunosorbent assay (ELISA) kits following the manufacturer’s protocols.

### 2.9 IHC analysis

IHC analysis was performed on paraffin-embedded skin tissue sections. Antibodies anti-Ki67 (1:500) and anti-PCNA (1:500) were used. The images of sections were photographed using a light microscope.

### 2.10 RNA sequencing

Skin tissues were extracted randomly from the control, IMQ, and LYS-High groups, respectively (*n* = 3). Total RNAs were extracted to generate cDNA libraries for Illumina Hiseq4000 sequencing (CA, United States). Before further analyses, we used the relative logarithmic expression boxplots and principal component analysis (PCA) to control the quality of the data. Gene ontology (GO) and Kyoto Encyclopedia of Genes and Genomes (KEGG) annotations with assembled differentially expressed genes (DEGs) were performed, and subsequently, LYS-induced DEGs and associated signaling pathways were generalized. Furthermore, quantitative polymerase chain reactions (qPCRs) and Western blotting (WB) were used to check the expression of some DEGs related to target signaling pathways to verify the results of RNA sequencing.

### 2.11 Real-time qPCR assay

Total RNA was extracted using TRIzol reagent (No. CR2112079; Servicebio). Then the RNAs were reverse transcribed into cDNA using a PrimeScript RT means Real time Master Mix (No. AL61314A, TaKaRa, Tokyo, Japan). qPCR was performed using TB green is a fluorescent dyes Green Premix Ex Taq Mremix Ex Taqme (TaKaRa) on a LightCycler480 system. The PCR cycle parameters were as follows: denatured at 95 °C for 30 s, followed by 45 cycles of 95°C for 5 s and 60°C for 30 s, and then followed by one cycle of 95°C for 5 s and 60°C for 60 s (melting). The data were analyzed using the 2^−ΔΔCT^ method. The primers used in this study are listed in Supplementary Table S1.

### 2.12 Western blotting

Approximately 100 mg of skin tissue was used to extract protein using 1 mL of RIPA buffer containing 10 μL of PMSF (No. KGP704/KGP704-100; Keygen Biotech, China). The protein concentration was determined using BCA assay (No. Abs9232; Absin, China), and total proteins were separated using 6%–12% SDS-PAGE. The separated proteins were then transferred to PVDF membranes and blocked with 5% BSA in TBST for 2 h. After blocking, the membranes were washed three times with TBST and incubated with primary antibodies including PCNA, p38, P-p38, MEK3 and MEK6 (1:1,000 diluted), GAPDH (1:2000 diluted), and Ki67 (1:800 diluted) overnight at 4°C. The incubation with secondary HRP-conjugated antibody (1:1,000 diluted) was then performed at room temperature for 1 h to detect antibody binding. The protein bands were quantified and analyzed using an Image J system (National Institutes of Health, Bethesda, MD, USA). Finally, the data were expressed as band intensity normalized to GAPDH or their respective total proteins.

### 2.13 Statistical analysis

The data were expressed as mean ± standard deviation (SD). A one-way analysis of variance was performed to analyze the data using SPSS Statistics 24.0 (IBM SPSS Inc., Chicago, USA). A *p*-value of <0.05 indicated a statistically significant difference.

## 3 Results

### 3.1 Quantification of bioactive metabolites

**FIGURE 1:**
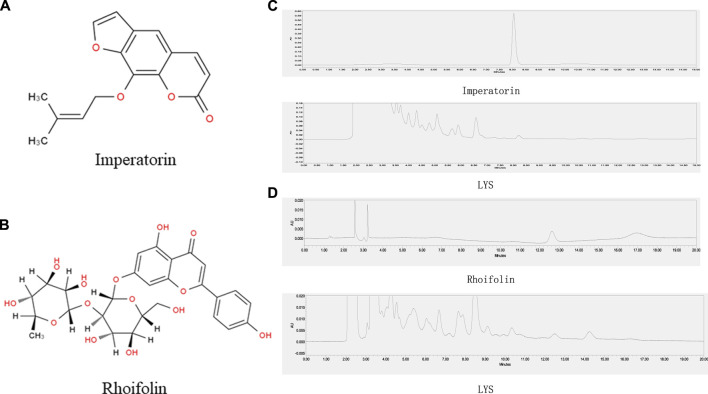
Structural formula **(A, B)** and HPLC of imperatorin and rhoifolin **(C, D)**. Retention time of imperatorin and rhoifolin were approximately 8.2 and 12.6 min, respectively.

Fengweicao and Baizhi are two major metabolites of the LYS. Studies showed that imperatorin, an active coumarin of Baizhi, could alleviate psoriasiform dermatitis by blocking neutrophil respiratory burst, adhesion, and chemotaxis *via* selective phosphodiesterase four inhibition (Tsai et al., 2021). On the contrary, rhoifolin is one of the main bioactive metabolites of Fengweicao, and is generally used for its quality control. Rhoifolin is a plant flavonoid known to have anti-inflammatory properties (Fang et al., 2020). Hence, we chose the active ingredient of Baizhi (imperatorin) and the active ingredient of Fengweicao (rhoifolin) to preliminarily establish quality control. The HPLC determinations for imperatorin and rhoifolin were validated; the chromatograms are shown in Figure 1. The results revealed good reproducibility and adequate stability. The recoveries complied with the requirements of Pharmacopoeia 2020. The methodological parameters and assaying results are listed in Supplementary Table S2.

### 3.2 LYS ameliorated psoriasis-like skin lesions in mice

**FIGURE 2:**
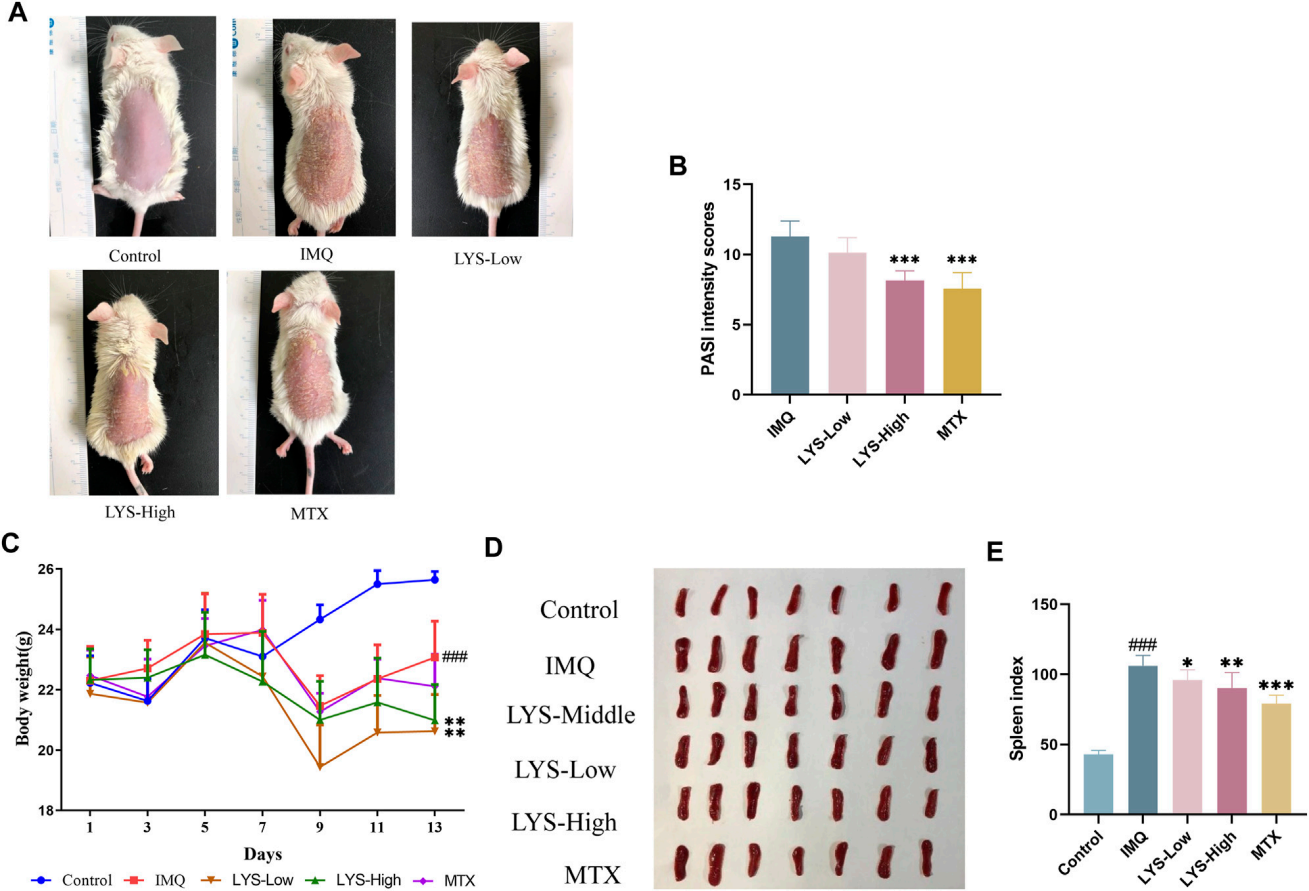
Effects of LYS treatment against psoriasis-like symptoms (*n* = 7). **(A)** Images of psoriatic skin lesions in five different groups. **(B)** PASI intensity scores of skin lesions in five different groups. **(C)** Changes in body weight in mice. **(D)** Spleen images and **(E)** cumulated spleen index in five different groups. ^###^ *p* < 0.001 vs. the control group; ^*^ *p* < 0.05, ^**^ *p* < 0.01, and ^***^ *p* < 0.001 vs. the IMQ group.

Typical signs of erythema, scaling, and epidermal thickening appeared after 5 days of IMQ application; the symptoms of psoriasis attenuated significantly with the treatment with LYS compared with that in the IMQ group (Figure 2A). The PASI intensity scores were determined for assessing lesion severity in all mice. The PASI intensity scores continually increased after the IMQ application compared with that in the control group. However, the PASI intensity scores decreased gradually after MTX or LYS (both LYS-Low and LYS-High groups) treatment. The PASI intensity scores significantly decreased in the LYS-High- and MTX-treated groups compared with the IMQ group (both had *p*-value < 0.001.) (Figure 2B and S1). During the experiment, significant differences in body weight were observed between the blank and IMQ groups (*p* < 0.001). After LYS treatment, the body weight decreased significantly compared with the model group (*p* < 0.01) (Figure 2C). The spleen index increased in the IMQ group, but apparently decreased in the LYS-High, LYS-Low, and MTX-treated groups (*p* < 0.01, *p* < 0.05, and *p* < 0.001, respectively) (Figures 2D,E).

### 3.3 LYS attenuated tissue pathology of psoriasis

**FIGURE 3:**
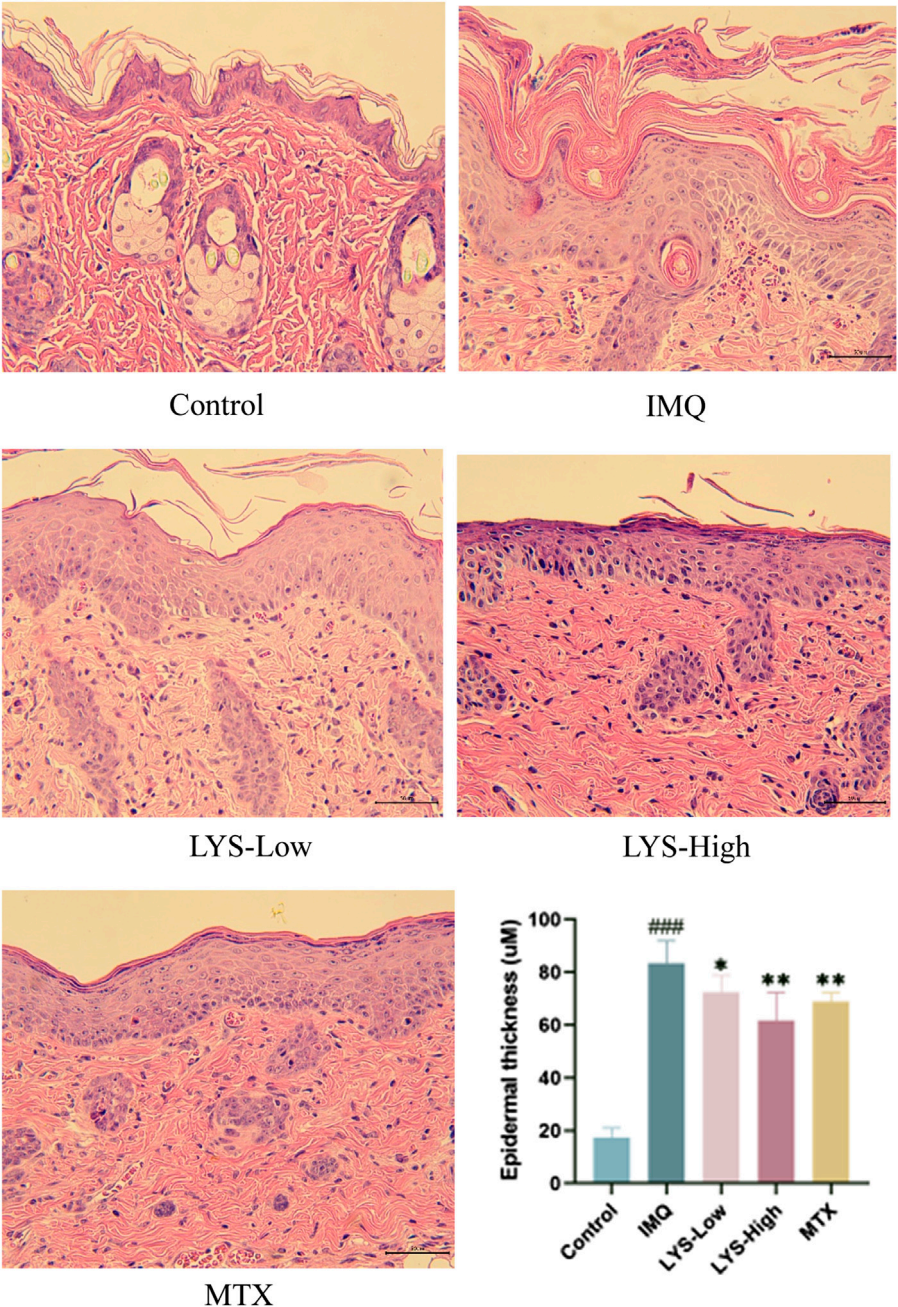
Histopathological images and epidermal thickness of different mice (images magnified × 100, *n* = 7). Images of H and E-stained sections were taken under a microscope to observe the epidermal thickness in different groups. Three fields were selected for each image, and every field was measured five times. ^###^ *p* < 0.001 vs. the control group; ^*^ *p* < 0.05, ^**^ *p* < 0.01, and ^***^ *p* < 0.001 vs. the IMQ group.

H and E staining revealed that the epidermal layer in the IMQ group was thickened, and numerous lymphocyte infiltrations were observed in the dermis and epidermis. After treatment with MTX and different doses of LYS, the thickness of the epidermal layer was effectively reduced, the stratum corneum became smooth, and the lymphocyte infiltration in the dermis layer was reduced. Furthermore, the higher the dose of LYS, the more obvious was the effect; notably, the high dose of LYS was equivalent to that of MTX (Figure 3).

### 3.4 LYS decreased inflammatory cytokines in the serum of mice with psoriasis

**FIGURE 4:**
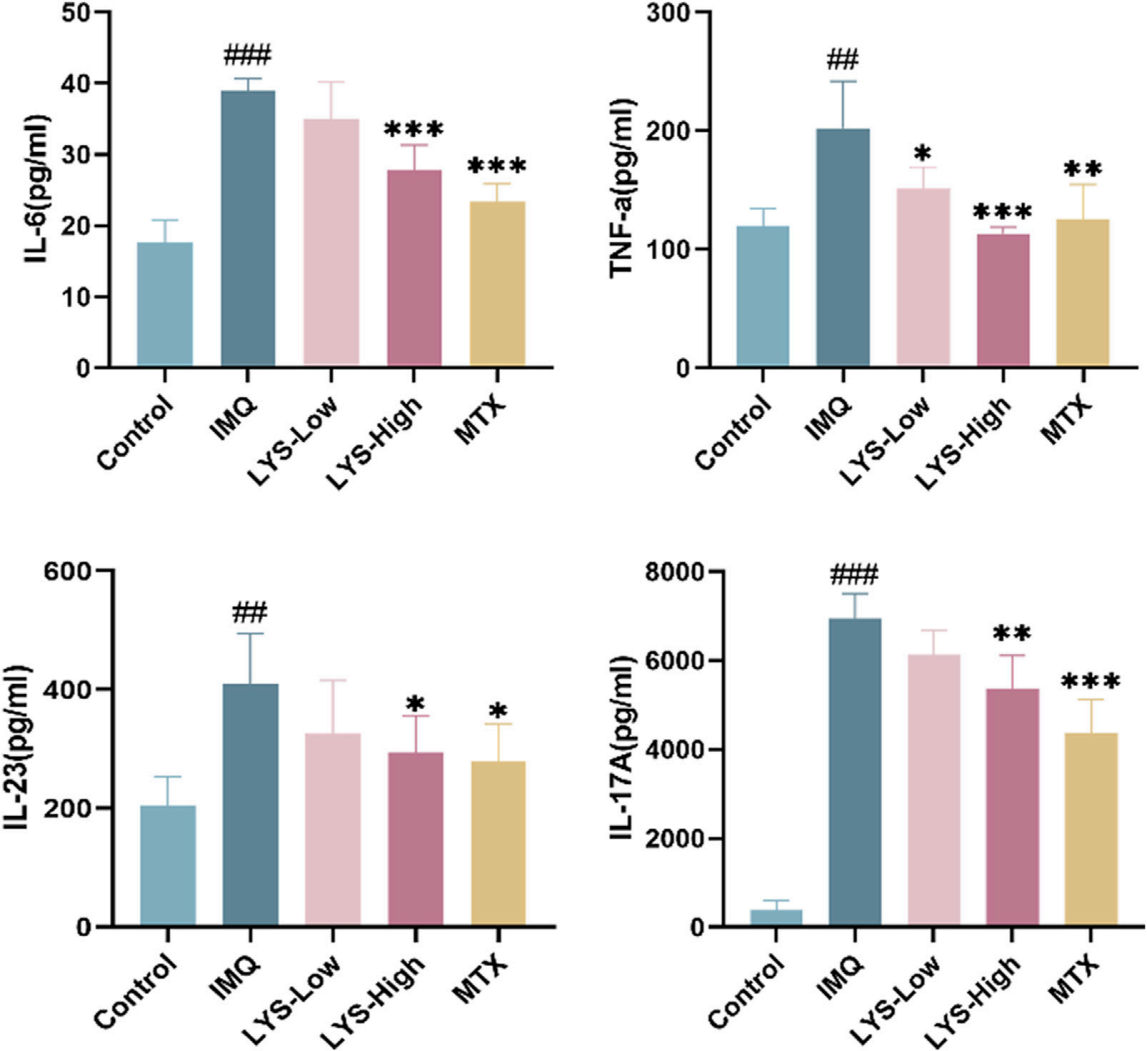
Effects of LYS on the serum levels of the inflammatory cytokines in IMQ-induced mice with psoriasis. IL-6, TNF-α, IL-23, and IL-17 levels were measured using ELISA. The data are expressed as mean ± SD (*n* = 7). ^##^ *p* < 0.01 vs. the control group; *p* < 0.05, ^**^ *p* < 0.01, and ^***^ *p* < 0.001 vs. the IMQ group.

The effects of LYS on serum cytokines were detected using ELISA. IL-6, TNF-α, IL-23, and IL-17 levels significantly increased in IMQ-induced mice with psoriasis (Figure 4; *p* < 0.01). The levels of these cytokines significantly decreased in the LYS and MTX groups compared with the IMQ group (*p* < 0.05). In the LYS group, the levels of IL-6, TNF-α, IL-23, and IL-17 decreased in a dose-dependent manner.

### 3.5 LYS reduced keratinocyte proliferation in mice with psoriasis

**FIGURE 5:**
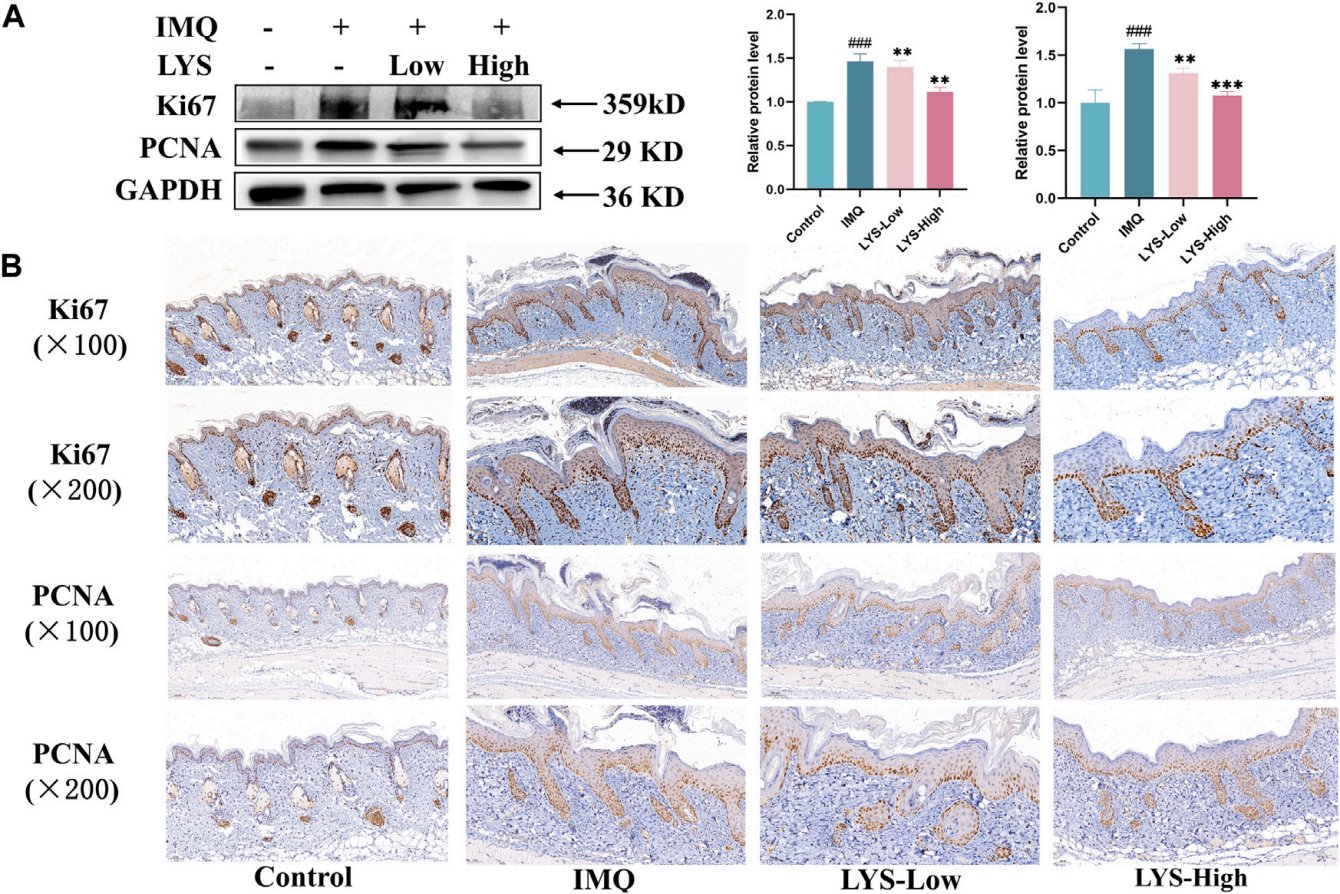
LYS decreased Ki67 and PCNA protein expression, as evident in WB **(A)** and IHC staining analyses **(B)**. The data are expressed as the mean ± SD (*n* = 3). ^###^ *p* < 0.001 vs. the control group; ^**^ *p* < 0.01, and ^***^ *p* < 0.001 vs. the IMQ group.

PCNA was used as an indicator to evaluate cell proliferation status as an integral aspect of cell DNA synthesis. Ki67 was a related antigen of proliferating cells, and its function was related to mitosis. PCNA and Ki67 were indispensable in cell proliferation. We used WB and IHC methods to detect these two indicators. The expression of PCNA and Ki67 in the skin tissues of mice treated with LYS was significantly reduced, especially in the LYS-High group (Figure 5A). The skin IHC results also confirmed that PCNA and Ki67 levels decreased in both LYS-Low and LYS-High groups (Figure 5B), suggesting that LYS treatment could reduce keratinocyte proliferation in mice with psoriasis.

### 3.6 RNA sequencing data analysis for quality control

High-throughput sequencing was used for the systematic analysis of gene expressions in the skin tissues of mice in the control, IMQ, and LYS-High groups. Approximately 23.99, 24.79, and 22.38 million raw reads were obtained from the control, IMQ, and LYS-High groups, respectively. The distribution of samples was examined *via* PCA to verify the rationality of the experimental design and the uniformity of biological duplicate samples (Supplementary Table S2). Correlation analysis revealed that the samples from the same group were distributed closely, and the LYS samples were closer to the control group than to the IMQ group, suggesting that the LYS group had a higher correlation with the control group than with the IMQ group (Supplementary Table S2).

### 3.7 Identification of DEGs

**FIGURE 6:**
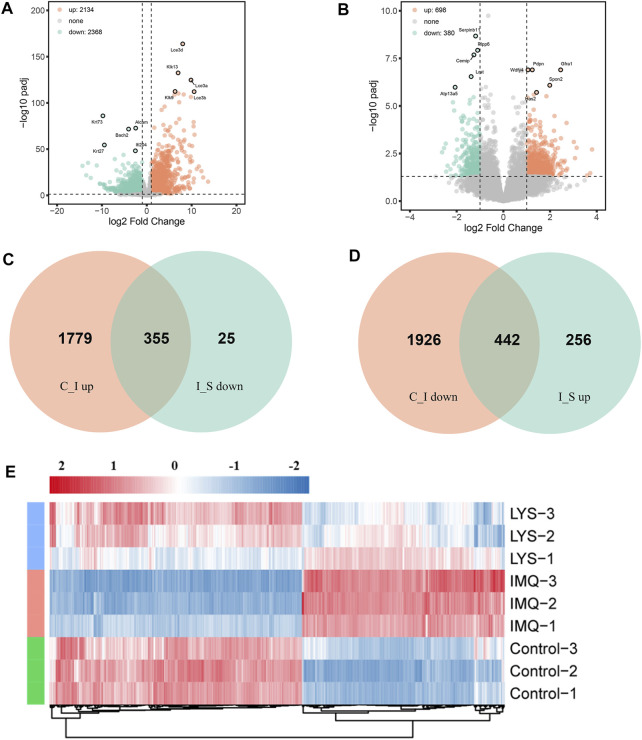
DEGs among the control, IMQ and LYS-High groups. **(A)** Volcano plot of DEGs expressed in the IMQ group compared with the control group. The horizontal axis indicates expression changes (log) of the genes in different treatment groups, while the vertical axis shows the differences in gene expression. The discrepancy was more significant with smaller *p* values and higher −log_10_ values (adjusted *p* values). Splashes were for different genes, among which gray dots were for genes with no significant discrepancy, orange dots were for genes significantly upregulated, and green dots were for significantly downregulated genes. **(B)** Volcano plot of DEGs expressed in the LYS group compared with the IMQ group. **(C)** Venn diagram showing that the expression of DEGs increased with IMQ treatment and then decreased with LYS treatment. **(D)** Venn diagram showing that the expression of DEGs decreased with IMQ treatment and then increased with LYS treatment. **(E)** Cluster assay was used to analyze the expression of 797 DEGs among the control, IMQ, and LYS groups.

A rigorous comparison at adjusted *p* < 0.05 and |log_2_FoldChange| > 0.58 was made to identify the number of DEGs for different groups. Hence, the gene expression analysis showed that 4,502 genes were significantly differentially expressed in the IMQ group compared with the control group, including 2,134 upregulated and 2,368 downregulated genes (Figure 6A), and 1,078 genes were expressed in the LYS group compared with the IMQ group, including 698 upregulated and 380 downregulated genes (Figure 6B). As depicted in the Venn diagram, the expression of 355 DEGs increased with IMQ treatment and then decreased with LYS treatment (Figure 6C), and the expression of 442 DEGs decreased with IMQ treatment and then increased with LYS treatment (Figure 6D). The heatmap of hierarchical clustering analysis was a useful tool to reveal the differences in the expression of differential probes intuitively. The similarity in the abundance profiles of differential probes was observed (Figure 6E), exhibiting a satisfactory discriminatory value among the control, IMQ, and LYS-High groups.

### 3.8 GO and KEGG pathway analysis

**FIGURE 7:**
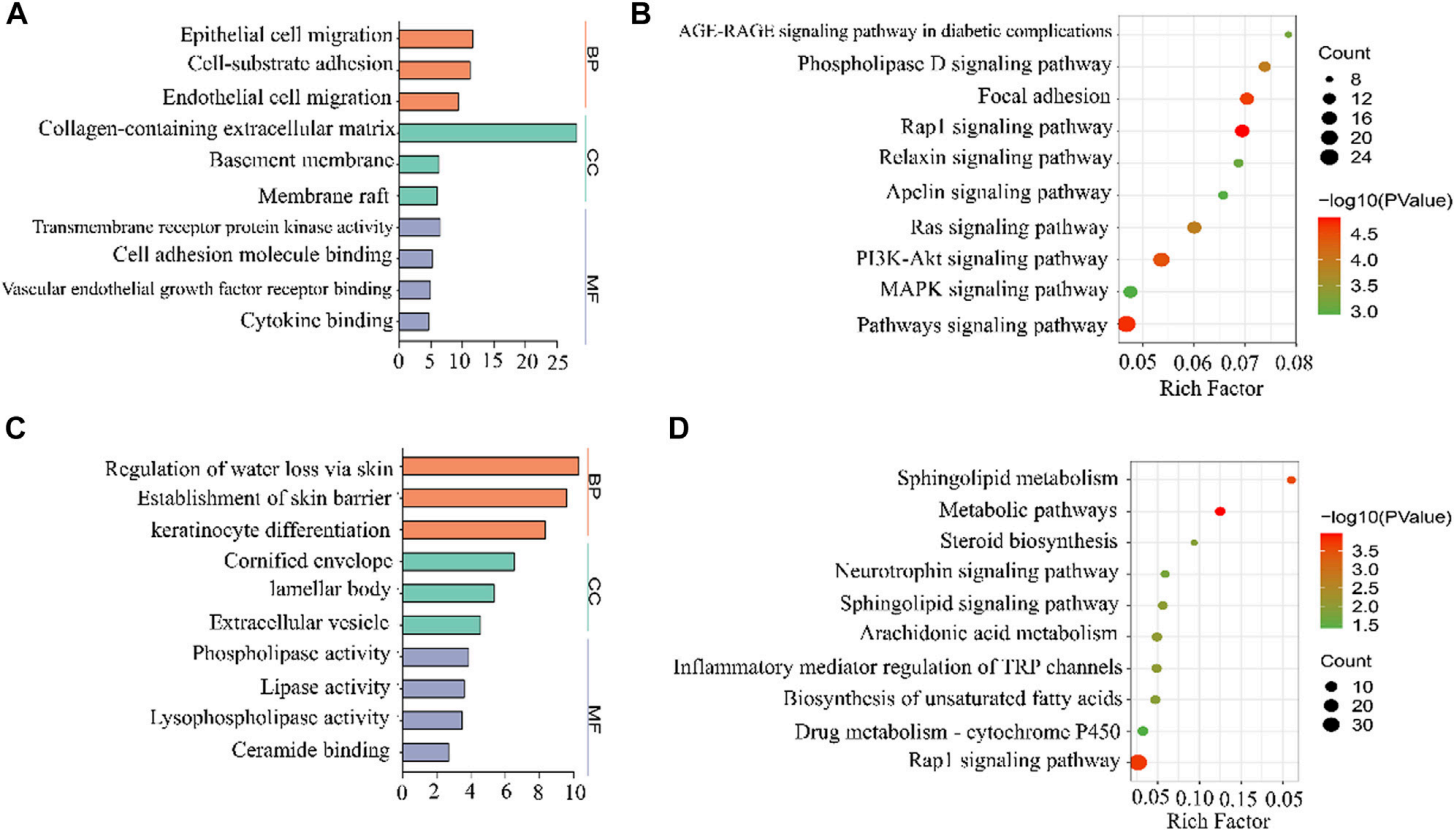
GO and KEGG analyses of DEGs among the control, IMQ, and LYS-High groups. GO enrichment analysis predicted the functional roles of target host genes based on the control, IMQ, and LYS-High groups. **(A)** Expression of DEGs decreased with IMQ treatment and then increased with LYS treatment. **(B)** Expression of DEGs increased with IMQ treatment and then decreased with LYS treatment. The top-three terms of BP, CC, and MF analyses are represented by three different colors in this figure: Orange, BP; green, CC; purple, MF. **(C)** KEGG enrichment analysis based on DEGs whose expression decreased with IMQ treatment and then increased with LYS treatment. **(D)** KEGG enrichment analysis based on DEGs whose expression increased with IMQ treatment and then decreased with LYS treatment.

We performed GO enrichment analysis of 442 DEGs whose expression decreased with IMQ treatment and then increased with LYS treatment and 355 DEGs whose expression increased with IMQ treatment and then decreased with LYS treatment based on the GO database. These DEGs were categorized into three main groups: biological process (BP), cellular component (CC), and molecular function (MF). These DEGs were linked to the keratinocyte adhesion, migration, and dysfunction based on BP subgroups (Figure 7A): “epithelial cell migration,” “cell-substrate adhesion,” and “endothelial cell migration,” which were the three most abundant GO terms within the BP category (442 DEGs), and “regulation of water loss *via* the skin,” “establishment of the skin barrier,” and “keratinocyte differentiation” (355 DEGs) (Figure 7C). In terms of CCs, these DEGs were enriched in “collagen-containing extracellular matrix,” “basement membrane,” “cornified envelope,” “lamellar body,” and so on. Moreover, DEG mRNAs were enriched in MFs such as “transmembrane receptor protein kinase activity,” “cell adhesion molecule binding,” and “lipase activity.”

The KEGG enrichment analysis led to the identification of the top 10 pathways, including “AGE-RAGE signaling pathway in diabetic complications,” “Rap1 signaling pathway,” “Ras signaling pathway,” “MAPK signaling pathway,” and so forth (Figure 7B and 7 days). Among all the pathways, the top-ranking enriched term was “Rap1 signaling pathway.” Interestingly, Rap1–MAPK signaling pathway was mostly associated with cell proliferation, which might play a key role in the abnormal proliferation of keratinocytes.

### 3.9 LYS inhibited the activation of Rap1–MAPK signaling pathways

**FIGURE 8:**
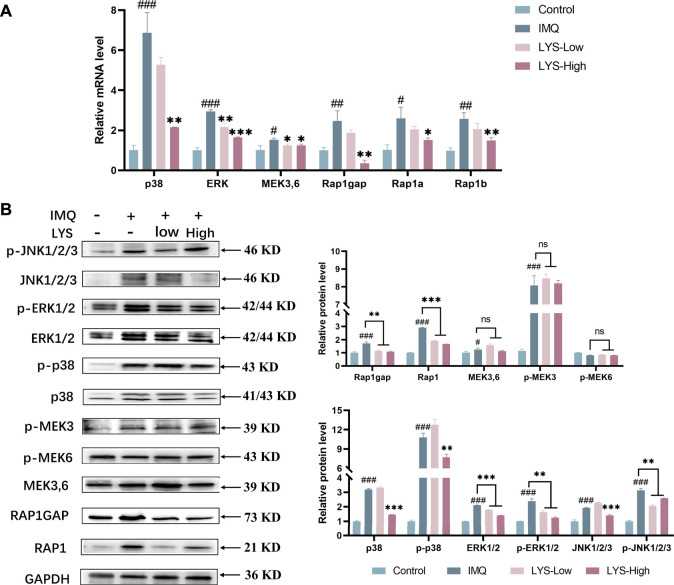
Effects of LYS on the expression of Rap1 (Rap1 and Rap1gap) and MAPKs (p38, JNK, ERK1/2, and MEK3,6) signaling pathways in the psoriasis-like mouse model. Expression of p38, ERK, MEK3/6, Rap1gap, Rap1a, and Rap1b mRNAs in the back skin tissues was detected using reverse transcription–polymerase chain reaction **(A)** and the relative protein expression was detected using WB **(B)**. The data are expressed as mean ± SD (*n* = 3). ^#^ *p* < 0.05, ^##^ *p* < 0.01, and ^###^ *p* < 0.001 vs. the control group; ^*^ *p* < 0.05, ^**^ *p* < 0.01, and ^***^ *p* < 0.001 vs. the IMQ group.

We further detected the expression of critical molecules at the mRNA and protein levels to further verify the role of Rap1–MAPK signaling pathways in the proliferation of psoriatic keratinocytes. As shown in Figure 8A, the mRNA expression of p38, ERK, MEK3, MEK6, Rap1gap, Rap1a, and Rap1b was upregulated in the IMQ group compared with the control group (*p* < 0.05, *p* < 0.01, and *p* < 0.001, respectively). LYS significantly suppressed the expression of the aforementioned targets (*p* < 0.05, *p* < 0.01, and *p* < 0.001, respectively).

As shown in Figure 8B, IMQ treatment significantly increased the MAPK-related protein expression and phosphorylation level of p38, ERK1/2, and c-Jun N-terminal kinase (JNK1/2/3) (*p* < 0.001). These IMQ-induced enhancements in the expression were markedly prevented by LYS in a dose-dependent manner except p-JNK (*p* < 0.01 and *p* < 0.001). The expression levels of MEK3/6 and p-MEK3 were increased by IMQ treatment (*p* < 0.05 and *p* < 0.001), but were not suppressed by LYS treatment. IMQ treatment upregulated the expression of Rap1 and Rap1gap, while LYS significantly downregulated their expression (*p* < 0.001 and *p* < 0.01).

## 4 Discussion

Psoriasis is a complex disease caused by different factors, and its exact pathogenesis is unknown. Previous studies showed that keratinocyte hyperproliferation might play a critical role in the initiation and maintenance of psoriasis (Ortiz-Lopez et al., 2022).

Recent studies reported that the theory of the “IL-23/Th17 axis” could explain the pathogenesis of psoriasis to some extent. Activated by IL-23 and sourced from dendritic cells and macrophages in the psoriatic dermis, Th17 and T cells release inflammatory cytokines such as TNF-α, IL-6, IL-17A, and IL-22 (Di Cesare et al., 2009; Ghoreschi et al., 2021). These inflammatory cytokines act on keratinocytes, resulting in epidermal hyperplasia, hyperkeratosis, and other typical pathological changes in psoriasis. In the inflammatory microenvironment of skin, keratinocytes produce more IL-23, chemokines, and other inflammatory factors, forming a positive feedback loop of the “IL-23/Th17 axis,” which amplifies and exacerbates the chronic inflammatory process of psoriasis (Deng et al., 2016; Ehst et al., 2021).

Cell cycle–related antigens Ki67 and PCNA are the most common proliferation markers in the IMQ-induced psoriasis-like mouse model. This study showed that the expression of Ki67 and PCNA was significantly upregulated in the model group, accompanied by an increase in “IL-23/Th17 axis”–related inflammatory factors, compared with that in the control group. This was consistent with the previous findings (Gangadevi et al., 2021; Kuai et al., 2021). After LYS intervention, the expression of the proliferating protein and the level of inflammatory factors in the treatment group decreased significantly. This indicated that LYS could reduce the proliferation of keratinocytes and improve the symptoms of psoriasis dermatitis by reducing the secretion of inflammatory factors and the expression of proliferative proteins in serum.

MAPK plays an important role in regulating most cellular processes, including proliferation, differentiation, and apoptosis and can be grouped into four subfamilies: ERK, p38, JNK, and BMK1 (also called ERK5) (Zhao et al., 2018; Guo et al., 2022). P38, ERK1/2, and JNK have potential pathogenic effects in the development of psoriasis (Sakurai et al., 2019). Among these, the activation of p38 in the skin is the key event of psoriasis lesions. P38 is highly activated in the epidermis of human psoriatic lesions (Johansen et al., 2005). Moreover, the role of the IL-17 pathway is considered to depend partly on the activation of p38 MAPK (Roussel et al., 2010). As previously discussed, previous studies reported an increase in the expression of p38, p-p38, ERK, p-ERK, JNK, and p-JNK in skin tissues in the IMQ group, and LYS alleviated the expression levels in the aforementioned targets.

p38 MAPK can be activated by Rap1, which can regulate cell growth and death (Gutiérrez-Uzquiza et al., 2010). Rap1 belongs to the Ras family of low-molecular-weight guanosine-5′-triphosphate enzymes. Rap1 has two isoforms: Rap1a and Rap1b, showing 95% identity. The Rap1 protein acts as a molecular switch by cycling between two states: the inactive GDP-binding form and the active GTP-binding form (Jaśkiewicz et al., 2018). A recent study showed that Rap1 also played an important role in inflammatory reactions. As the basic tool of the immune system, the expression of leukocytes in psoriatic lesions increases significantly (Capriotti et al., 2018). Integrin is mainly responsible for leukocyte adhesion. Rap1 is considered the key medium for CD31 (an important integrin adhesion amplifier) cytoplasmic tail to transmit the signal-inducing T cell adhesion through α4β1 (vla-4) and β2 (lfA-1) integrins (Reedquist et al., 2000). Thus, a study revealed that CD31 selectively activated Rap1, and the activated Rap1 mutant stimulated the adhesion of T cells to intercellular adhesion molecule 1. These data suggested that Rap1 played a key role in leukocyte adhesion, which allowed the progression of inflammatory response. In agreement with previous studies, IMQ application increased inflammation signaling cascades. Molecularly, LYS treatment reduced the expression of Rap1 and Rap1gap.

The findings of this study demonstrated that LYS primarily targeted keratinocytes *in vivo* by inducing the secretion of “IL-23/Th17 axis”–related inflammatory factors, thereby reducing the hyperproliferation of keratinocytes. The results of RNA sequencing and molecular biology experiments confirmed that the antipsoriatic effect of LYS might be related to the inhibition of Rap1–MAPK signaling pathway activation.

## 5 Conclusion

The results of this study revealed that LYS had obvious anti-psoriatic effects in the mice with IMQ-induced psoriasis. The anti-psoriasis effect of LYS was related to the inhibition of inflammatory factor secretion, keratinocyte proliferation, and the activation of the Rap1–MAPK signaling pathway. These findings confirmed the therapeutic effect and mechanism of LYS in treating psoriasis to some extent, thus providing important evidence for the clinical application of LYS.

## Data Availability

The original contributions presented in the study are included in the article/Supplementary Material. The RNA-sequencing data are deposited in the GEO repository, accession number GSE226452.
