# Supplementary material for: Effect and mechanism of longkui yinxiao soup in treating psoriasis in mice
Source: Front Pharmacol. 2023 Mar 13;14:1136604. doi: 10.3389/fphar.2023.1136604 (PMC10040526; doi:10.3389/fphar.2023.1136604)
Supplement: Supplementary file 1 [file DataSheet1.docx]

Supplementary Material

**Effect and mechanism of Longkui Yinxiao Soup in treating psoriasis in mice**

**Congcong Zhu^1,2^†, Ya Chen^1,2^†, Zongguang Tai^1,2^, Huijun Pan^1,2^, Min Shen^1,2^, Zhongjian Chen^1,2^* and Quangang Zhu^1,2^***

***Corresponding Author:**

Dr. Quangang Zhu

Email: [qgzhu@126.com](mailto:qgzhu@126.com)

Dr. Zhongjian Chen

Email:aajian818@163.com

# Supplementary Figures and Tables

Supplementary Table S1 Primers used for qPCR assays

| Gene | Forward primer | Reverse primer |
| --- | --- | --- |
| GAPDH | TGGGAAGCTGTGGCGTGAT | TCAGATCCACAACCGACACATT |
| ERK | ATCTCAACAAAGTTCGAGTTGC | GTCTGAAGCGCAGTAAGATTTT |
| MEK3,6 | ACTATCGGAGACAGAAACTTCG | CACTTTCTCTACCACCCCATAG |
| p38 | AGGAATTCAATGACGTGTACCT | AGGTCCCTGTGAATTATGTCAG |
| Rap1gap | CTCCCACCCTGCTTCACCCTAC | CGCTGTTCATCCATCCTGCTTCC |
| Rap1a | AGATGGCGGACACTCTTCTCAGG | CTGCTGGTGCTGGAGGAGGAG |
| Rap1b | GAACGGACAAGGCTTCGCTCTG | CCAAGTCGCATTTATTGCCAACCAG |

Supplementary Table S2 The methodological parameters and assaying results

| Bioactive  components | Regression equation of  standard curve | Precision  RSD (%) | Reproducibility  RSD (%) | Stability  (24h) | Recovery  （%） | Content  (ug·g-1) |
| --- | --- | --- | --- | --- | --- | --- |
| Imperatorin | Y=25150X-2958.1, r=1 | 0.47 | 0.55 | 1.45 | 96.45 | 71.63 |
| Rhoifolin | Y=23613X-2886.3, r=1 | 0.30 | 2.16 | 2.27 | 88.95 | 25.93 |


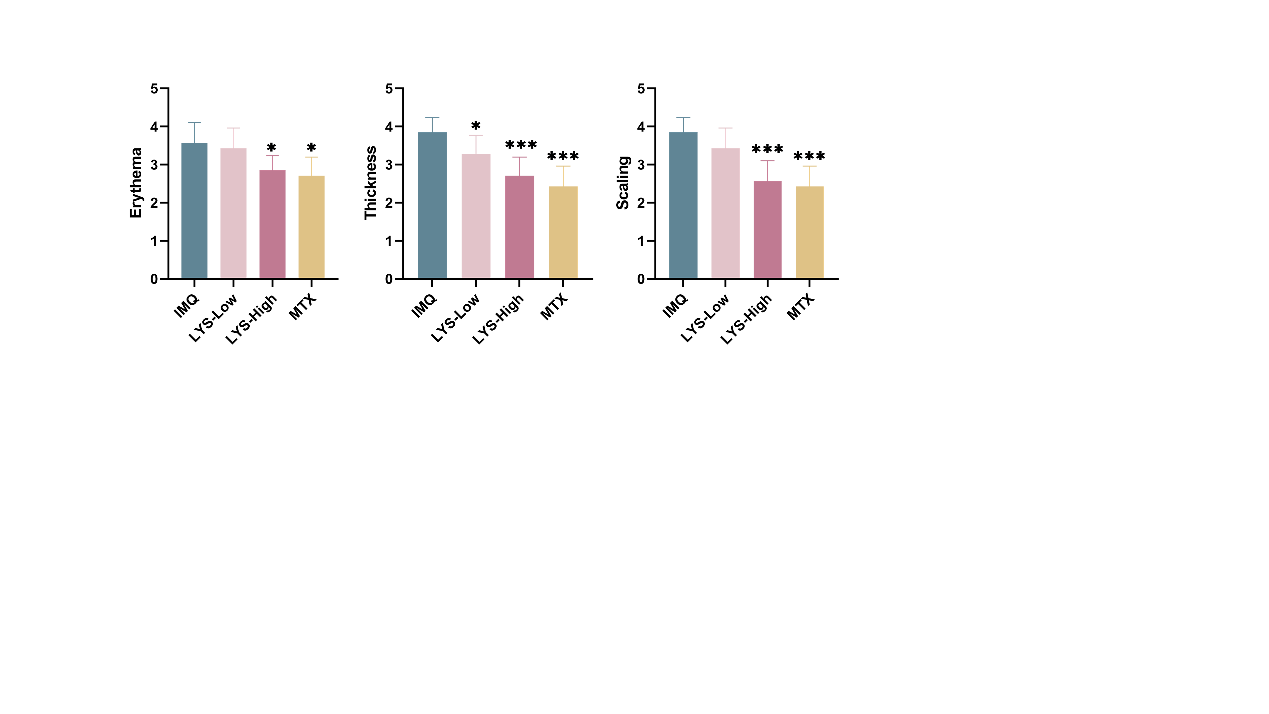


Supplementary Figure S1：PASI intensity scores (erythema, scaling, epidermal thickness) of skin lesions in five different groups.


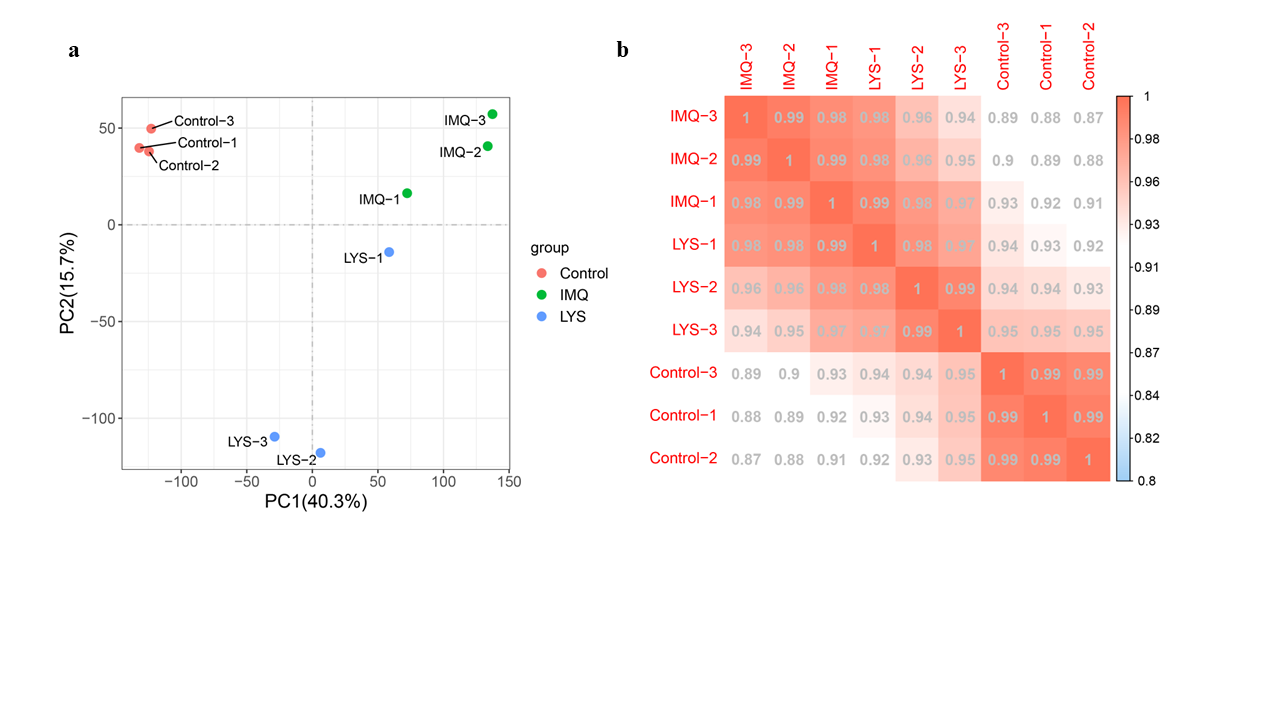


Supplementary Figure S2: PCA and Cor analysis based on the microarray data. (a) The distribution of DEGs for the samples from control, IMQ and LYS groups in PCA. (b) Control, IMQ and LYS groups in Cor analysis
